# Return-to-activity after anatomical reconstruction of acute high-grade acromioclavicular separation

**DOI:** 10.1186/s12891-016-0989-8

**Published:** 2016-04-02

**Authors:** T. Saier, J. E. Plath, K. Beitzel, P. Minzlaff, J. M. Feucht, S. Reuter, F. Martetschläger, Andreas B. Imhoff, M. Aboalata, S. Braun

**Affiliations:** Department of Orthopedic Sports Medicine, Technische Universität München, Munich, Germany; Department of Reconstructive Joint Surgery, Berufsgenossenschaftliche Unfallklinik Murnau, Murnau, Germany; Chirurgische Klinik Dr. Rinecker, Munich, Germany; Department Orthopedics and Traumatology University of Freiburg, Freiburg, Germany; Center for Shoulder and Elbow Surgery, ATOS Clinic Munich, Munich, Germany; Department of Orthopaedic Surgery, Mansoura University, Mansoura, Egypt

**Keywords:** Acromioclavicular separation, Rockwood V, Acromioclavicular, Reconstruction, Arthroscopy, Tight-rope, suture button device, Return-to-activity, Returnto-sports

## Abstract

**Background:**

To evaluate return-to-activity (RtA) after anatomical reconstruction of acute high-grade acromioclavicular joint (ACJ) separation.

**Methods:**

A total of 42 patients with anatomical reconstruction of acute high-grade ACJ-separation (Rockwood Type V) were surveyed to determine RtA at a mean 31 months follow-up (f-u). Sports disciplines, intensity, level of competition, participation in overhead and/or contact sports, as well as activity scales (DASH-Sport-Module, Tegner Activity Scale) were evaluated. Functional outcome evaluation included Constant score and QuickDASH.

**Results:**

All patients (42/42) participated in sporting activities at f-u. Neither participation in overhead/contact sports, nor level of activity declined significantly (n.s.). 62 % (*n* = 26) of patients reported subjective sports specific ACJ integrity to be at least the same as prior to the trauma. Sporting intensity (hours/week: 7.3 h to 5.4 h, *p* = .004) and level of competition (*p* = .02) were reduced. If activity changed, in 50 % other reasons but clinical symptoms/impairment were named for modified behavior. QuickDASH (mean 6, range 0–54, SD 11) and DASH-Sport-Module (mean 6, range 0–56, SD 13) revealed only minor disabilities at f-u. Over time Constant score improved significant to an excellent score (mean 94, range 86–100, SD 4; *p* < .001). Functional outcome was not correlated with RtA (n.s.).

**Conclusion:**

All patients participated in sporting activities after anatomical reconstruction of high-grade (Rockwood Type V) ACJ-separation. With a high functional outcome there was no significant change in activity level (Tegner) and participation in overhead and/or contact sports observed. There was no correlation between functional outcome and RtA. Limiting, there were alterations in time spent for sporting activities and level of competition observed. But in 50 % those were not related to ACJ symptoms/impairment. Unrelated to successful re-established integrity and function of the ACJ it should be considered that patients decided not return-to-activity but are very content with the procedure.

## Background

Acute separations of the acromioclavicular joint (ACJ) are common in young athletes [11]. In a large closed longitudinal cohort study in military cadets the incidence of ACJ injury was reported with 9.2 injuries per 1000 person-years [14]. Overall, most injuries occur in the third life decade [15]. Male individuals have been described to have a 2–8 times higher risk for ACJ injury compared to females [13, 14]. High-risk sports, with frequent checking of players and forceful contact to the ground, as in contact sports and martial arts have been shown to increase the risk for ACJ injury [14].

There is profound data on the satisfying short- and intermediate term clinical outcome after anatomical ACJ reconstruction in high-grade ACJ separation [1, 17, 18, 21].

Concerning return-to-sports only general data on an average time lost to injury of 2.5 months exists [14]. Little is known about precise return-to-activity (RtA) after anatomical ACJ reconstruction of acute complete separation. Proper information of the patient should be based on more detailed data, since unrealistic expectations may leave the patient dissatisfied even if the surgical procedure objectively was successful.

The purpose of this study was to evaluate sporting activity after arthroscopically assisted anatomical reconstruction of acute complete ACJ separation with two anatomic suture button devices. The primary hypothesis of this study was that anatomical ACJ reconstruction allows RtA to sporting activities as performed prior to injury. Secondary it was hypothesized, that participation in overhead and contact sports was not impaired at least 24 months after surgery.

## Methods

### Patient selection & study design

Between January 2007 and July 2011 a total of 49 consecutive isolated acute high-grade ACJ-separations (Rockwood Type V) were surgically treated in 49 individuals. This retrospective clinical study was conducted to assess return-to-activity for these patients using the institutional prospective research database.

Inclusion criteria for enrollment were: Radiological diagnosis of primary acute complete ACJ separation Rockwood Type V, treated with arthroscopically assisted anatomical ACJ-reconstruction with two independent suture-button devices, patient age 18–45 years, minimum follow-up of 24 months, and participation in sporting activities before trauma. Exclusion criteria were: Concomitant and/or other shoulder pathology (e.g. fracture, dislocation, rotator-cuff-, SLAP-lesion) with ACJ separation at time, time from ACJ separation to surgery >4 weeks, ACJ separation type Rockwood I-IV, additional CC-ligament augmentation with hamstring-tendon autograft, secondary previous and/or meanwhile history of shoulder condition/surgery until follow-up, and no participation in sporting activities before index trauma.

### Operative technique and postoperative treatment

Arthroscopically assisted anatomical ACJ reconstruction, using two independent suture-button devices (TightRope, Arthrex, Naples, FL, USA), was performed as described by Walz et al. [22]. In short: diagnostic glenohumeral arthroscopy was performed to rule out any concomitant pathology. Three standard arthroscopy portals (posterior, anterior, and anterolateral) and a 2–3 cm incision perpendicular to the clavicle about 3.5 cm medial to the ACJ were performed. Under arthroscopic and radiologic visualization a 2.4 mm guide wire, using a standard ACJ drill guide (Arthrex, Naples, FL, USA), was drilled through the center of the clavicle, about 45 mm medial from the lateral clavicular edge, perforating the base of the coracoid posteriorly about 5 mm lateral to the medial border (conoidal position). A second drill guide wire was placed about 25 mm medial to the lateral clavicular edge (trapezoidal position). After fluoroscopic control, both guide wires were over-reamed with a 4.0 mm cannulated drill (Arthrex, Naples, FL, USA). Subsequently the suture-button devices were pulled in and both inferior buttons were flipped. Then sutures were tightened over the two clavicular buttons and tied to complete the procedure in a fluoroscopically controlled anatomical reduced position. The deltotrapezoidal fascia was thoroughly reconstructed, before the incision was closed in standard fashion.

Initial postoperative treatment consisted of limited ROM under instruction of a physical therapist and immobilization of the upper extremity with a standard armsling for 6 weeks for protection. Afterwards free ROM was allowed, unlimited activities of daily living were advised not before 12 weeks, and return to overhead and/or contact sports not earlier than 6 months after surgery.

### Outcome assessment

The side of surgery, arm dominance, sex, age, body mass index (BMI), and time since surgery (follow-up), as well as the preoperative Constant & Murley Score [2] was assessed.

At follow-up, patients were re-assessed by the Constant & Murley Score [2] and completed a sport specific questionnaire. The type of sporting activity before trauma and at follow-up was evaluated including 32 sporting and recreational activities as previously described [8, 12, 16]. Activity level was assessed using Tegner activity scale [19] and Disabilities of the Arm Shoulder and Hand (DASH) Sport Modul [4]. In addition Quick Disabilities of the Arm Shoulder and Hand (QuickDASH) questionnaire was used to assess overall shoulder function at follow-up [4].

Further patients were asked to report weekly participation in sports (sessions/week and hours/week) and level of competition (“recreational”, “competitive”, “professional”).

If sporting activity changed between the *in sana* status before injury and at follow-up, patients were asked for reasons for modified behavior: (I) “Shoulder-symptoms at the index side (e.g. pain, instability, limitations in ROM)”, or (II) “concerns about new trauma”, or (III) “other reasons then directly associated with the injury/procedure (e.g. change of activities in daily living due to social and/or professional reasons, shift in personal interests, time, self motivation)”. Multiple answers were possible.

Ability to participate in sports at follow-up compared to the *in sana* status before injury was evaluated with a subjective non-evaluated grading scale (“significant impaired”, “impaired”, “equivalent”, “improved”, significant improved” = I°-V°).

Finally, patients were asked with two closed questions (yes/no), if they felt in general capable to participate in overhead and/or contact sports at follow-up regardless of objective participation.

### Statistical analysis

SPSS Statistics 19 for Windows (IBM SPSS Statistics, New York, USA) was used for analysis. Descriptive statistics were calculated as means/median and standard deviations or frequencies. For comparison of preoperative and postoperative data, the paired-samples *t* test was used with a significance level set at .05. Correlations were calculated using Spearman’s correlation coefficient. Level of significance was set to *p* < .05. No power analysis was conducted for this study.

## Results

### Demographics

At a mean follow-up of 31.3 months (range 24–61 m) 42 individuals (39 m/3f) with 42 anatomical ACJ reconstructions after high-grade ACJ separation Rockwood Type V were enrolled in this retrospective study (86 % follow-up-rate; 6 patients were lost to follow-up and 1 experienced a new trauma to the ACJ that required revision surgery). At surgery mean age was 34.5 years (range 18–45y) and mean BMI was 25.9 (range 20.1–31.6). In 55 % (*n* = 23) surgery was conducted to the left side, with 93 % (*n* = 39) patients reporting on right arm dominance.

### Functional shoulder outcome

Mean preoperative Constant score after the injury was 36 (range 12–90, SD 17) vs. 94 (range 86–100, SD 4) at follow-up (*p* < .001). At follow-up mean QuickDASH was 6 (range 0–54, SD 11).

### Sporting activity

At follow-up all patients (42/42) participated in sporting activities at least occasionally (see Table 1). Before trauma and at follow-up median Tegner score was 7 (range 2–9, SD 1.6). Mean DASH Sport Module was 6 (range 0–56, SD 13) at follow-up. 62 % (*n* = 26) of the patients reported subjective sports specific ACJ integrity to be at least the same as prior to the trauma (see Table 1).

**Table 1:** Subjective sports specific ACJ integrity at follow-up (≥24 months after surgery) compared to the status prior to high-grade ACJ separation

Subj. sports specific ACJ integrity	At follow-up (≥24 m)	
	Total	%
Sign. impaired	1	2
Impaired	15	36
Same then previous	24	57
Better than previous	2	5
Sign. better than previous	0	0

### Type of sport

**Fig. 1:**
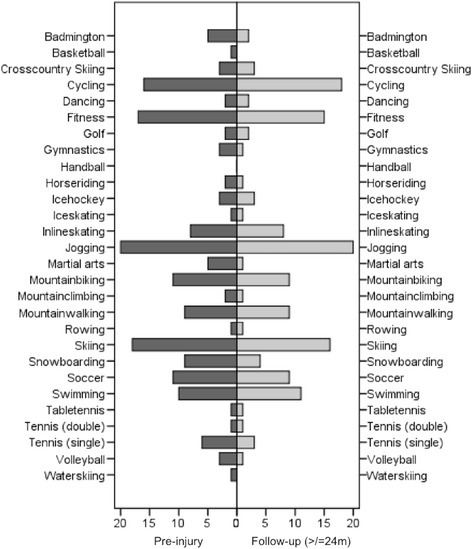
Sport specific return-to-activity after high-grade ACJ separation (Rockwood Type V): Left side participation before the trauma, right side participation at follow-up ≥24 months after surgery

The five most common sports performed at follow-up were: Jogging (48 %, *n* = 20), cycling (43 %, *n* = 18), skiing (38 %, *n* = 16), fitness (36 %, *n* = 15), and swimming (26 %, *n* = 11). The most common high-risk sports performed at follow-up were mountainbiking and soccer (each 21 %, *n* = 9). The general participation in different types of sport is summarized in Fig. 1 and Table 2.

**Table 2:** Change in sporting disciplines ≥24 months after anatomical ACJ reconstruction. Change in overhead and/or contact sports was not significant (n.s.). Inclusion criteria was ≥6 participants/discipline before trauma. (n.c.: no change)

Sporting Activity	Change Participation [%]
Snowboarding	−56 %
Tennis -single-	−50 %
Soccer	−18 %
Mountainbiking	−18 %
Fitness	−12 %
Skiing	−11 %
Inlineskating	n.c.
Jogging	n.c.
Trekking	n.c.
Swimming	10 %
Cycling	12 %
Contact sports	−2.9 %
Overhead sports	−3.3 %

Comparison of overhead sports, respectively contact sports performed prior to trauma vs. participation at follow-up showed a non significant decline in RtA (−2.9 %, n.s.; respectively −3.3 %, n.s.).

In general, 69 % of the patients felt capable to participate in overhead sports at follow-up. In 84 % the same was true for potentially participating in contact sports.

### Level of competition

The majority of individuals participated in recreational sports before trauma (71 %, *n* = 30) and at follow-up (83 %, *n* = 35). This change was significant (*p* = .02, see Table 4). At follow-up 62 % (*n* = 26) reported to perform sport activities at least on the same competition level as before the injury happened (details see Table 3).

**Table 3:** Competition level before high-grade ACJ separation (Rockwood Type V) vs. status at follow-up (≥24 months after surgery)

Competition level	Before Trauma		At follow-up (≥24 m)		Change
	Number	%	Number	%	%
Recreational	30	71	35	83	12*
Competitive	9	21	6	14	−7
Professional	3	7	1	2	−5

Changes were significant toward recreational level of competition (*p = .02)

### Sporting frequency

Before trauma patients reported a mean weekly participation in sports of 7.3 h (range 2–20, SD 4.5), that declined significantly to 5.4 h per week (range 1–12, SD 2.8) at follow-up (*p* = .004). The majority of patients participated in at least 3 sports sessions per week (78 %, *n* = 33) before the trauma. At follow-up this declined to 64 % (*n* = 27, n.s.). For details see Table 4.

**Table 4:** Sporting sessions per week before high-grade ACJ separation (Rockwood Type V) vs. weekly participation at follow-up (≥24 months after surgery)

Sporting Sessions	Before Trauma		At follow-up (≥24 m)		Change
	Total	%	Total	%	%
no-sports	0	0	0	0	0
<1×/w	1	2	3	7	5
1×/w	2	5	4	10	5
2×/w	6	14	8	19	5
3×/w	17	40	16	38	−2
4×/w	9	21	7	17	−5
5×/w	2	5	2	5	0
6×/2	1	2	1	2	0
daily	4	10	1	2	−7

### Evaluation of change in sporting activity

Patients with a change in sporting activity answered the questions for the reason why participation in athletic activities was altered compared to pre-injury condition 14 times with yes because of symptoms/impairment directly referred to the trauma/procedure. 7 times fear of a new trauma, and 7 times other reasons than the trauma/procedure itself (e.g. occupation, family, change in interests, time, self motivation) were named for changes in sporting activity.

### Correlation of functional shoulder outcome and sporting activity

At follow-up there was no correlation observed between functional shoulder outcome (Constant Score) and sporting activity (Tegner Scale), sporting frequency (hours/week), or ability to participate in pre-trauma sporting activities (n.s.).

## Discussion

The most important finding of this study is that all patients were able to participate in sporting activities after anatomical reconstruction of high-grade ACJ separations. Tegner activity scale did not differ between pre-injury level and at follow-up. There was no significant change in participation in overhead and/or contact sports observed. DASH Sport Module results showed only minor disabilities in sporting activities. The Constant Score increased significant and showed excellent average results at follow-up with no correlations to RtA. Since this study revealed significant decreases in sporting intensity and level of competition at follow-up there are limitations to the primary hypothesis. The secondary hypothesis was proofed, since no changes in overhead/contact sports were observed.

All patients included in this study have been treated with an arthroscopically assisted anatomical ACJ reconstruction using two independent suture-button devices as previously described [22]. Several studies have shown the satisfying short- and midterm clinical outcome of this procedure [17, 18, 21]. The Constant and QuickDASH scoring in this study -resembling only minor disabilities- is in support of these findings. Besides this technique many other surgical treatments for ACJ separation have been described, but not one technique has been shown to be clinically superior over the others [1, 9]. For acute complete ACJ separations implantation of a hook plate is still a concurring and frequently used technique. Others have investigated the pros and cons of the two techniques. The comparison of minimal invasive arthroscopically assisted ACJ reconstruction and implantation of a hook plate unveiled no significant differences on short-term clinical outcome [6]. With respect to RtA the authors of this study were not able to identify a comparable study conducted with hook plate treatment. Thus the authors of this study are not able to comment on the potential superiority of one technique over the other in terms of RtA after acute surgical treatment in high-grade ACJ separation. In general the authors are not aware of any study focusing on RtA after complete ACJ injury going in to more details than commenting unspecific on the subject [23]. On a register base, different studies analyzed incidence and time lost to return-to-play after ACJ injury in specific collectives, such as: US Military cadets, rugby, hockey, and football players [3, 5, 7, 10, 14]. Inclusion criteria, as well as treatment were heterogeneous and not conclusive (e.g. conservative vs. (prolonged) surgical treatment, approach to surgical treatment concerning indication and timing of surgery, as well as operative technique performed).

The majority of patients in this study participated in typical local sporting activities before trauma and also at follow-up. Asking for the reason to change sporting activities, 50 % of the patients named other reasons then direct impairing clinical symptoms. The authors hypothesize, that in a young and active population, especially in the age group of 30+, other factors (e.g. competing occupational and/or social interests advancing age, time, self motivation) may have affected sporting activities and level of competition substantially. This may be supported by the finding, that hours spent to participate in sports and number of sport sessions per week declined significantly. Functional outcome was high and there was no correlation with the investigated dimensions of sporting activity. In 2/3 of the study group the subjective functional outcome of the procedure was reported to be at least equivalent to the status prior to trauma. Patients felt capable to participate in overhead sports in 69 % and contact sports 84 %, although they did not necessarily participate in such activities prior to injury. Further, a recent study that investigated on return-to-sport after arthroscopic Bankart repair [20] revealed, that fear of re-injury, shifts in priority, mood, social support, and self motivation had a great influence on the decision to return-to-sport to the preinjury level. In this study, despite of a high level of shoulder function and subjective satisfaction with the procedure, a high number of patients choose not to return-to-sports for the above-mentioned reasons. In the opinion of the authors the same is true for anatomical reconstruction after high-grade ACJ. External and internal sources contribute to the decision to return-to-activity. Thus the authors hypothesize, that anatomical reconstruction after high-grade ACJ separation may have a higher potential for RtA as the actual results of the index study have shown.

Unrealistic expectations may lead to dissatisfaction despite successful surgical intervention. The authors strongly believe, that high-demanding individuals with high-grade ACJ separation should be informed carefully, that even in case of successful surgical treatment, RtA might be limited.

The study has it’s strengths with a strictly selected patient collective and exclusive focus on RtA after one surgical procedure in isolated acute high-grade ACJ separation (Rockwood Type V). This should limit bias of the results by excluding any concomitant shoulder pathologies and variability due to different surgical techniques.

But there are also several limitations of this study. The small total number of patients and thus even smaller numbers in specific activities may be misleading. Therefore the authors have grouped activities to overhead and contact sports in general to raise the value of the results. Further, change in participation is only represented for disciplines with at least six participants prior to injury. The authors believe that this approach allows a better interpretation of the case series. Further the geographic location of the studycenter may have influenced the type of sporting activities substantially. Preliminary identified high-risk activities from other studies (eg. rugby, american football, lacrosse, and hockey) are obviously underrepresented in this study and therefore the results may be not applicable for all populations. Overhead sports including extensive throwing (e.g. baseball) must be considered as underrepresented in this study for the same reasons as well. Further, 93 % of the individuals in this study group were right arm dominant, while surgery was performed on the left side in 55 %. The authors hypothesize, that this may bias the results particularly for overhead sports involving extensive throwing (e.g. Tennis, Badminton, Baseball). With the presented data the authors were not able to answer this specific question. There were only *n* = 3 professional athletes included in this study, thus the results of this study are thought to be less transferable and applicable to patients who are paid to return-to-activity. Last but not least nowadays in Europe early surgery for reducing and stabilizing acute high-grade ACJ is widely recommended to allow functional scarring of the disrupted ligaments. This might be contrary to a postponed surgical approach including autologous or allograft tendon reconstruction for a “chronic” situation (>3–6 weeks after trauma). Obviously participation bias exists because patients who declined to participate have different perspectives than patients that choose to participate in this study. Despite these limitations the data in the presented study represents a complete and current data set on return-to-activity after anatomical ACJ reconstruction in high-grade ACJ separation.

## Conclusion

All patients participated in sporting activities after anatomical reconstruction of high-grade ACJ-separation. With a high functional outcome there was no significant change in activity level (Tegner) and participation in overhead and/or contact sports observed. There was no correlation between functional outcome and RtA. Limiting, there were alterations in time spent for sporting activities and level of competition observed. But in 50 % those were not related to ACJ symptoms/impairment. Unrelated to successful re-established integrity and function of the ACJ it should be considered that patients decided not return-to-activity but are very content with the procedure.

## Ethics approval and consent to participate

IRB approval was obtained by the Ethics Committee of the Faculty of Medicine of the Technische Universität München, Munich, Germany (ID number: 76/14). Written consent to participate in the study was provided by every patient.

## Availability of supporting data

The dataset supporting the conclusions of this article is not available in an open access repository because it is part of an institutional dataset that is still under use. If there is interest in exploring specific issues, please contact the corresponding author (ABI).

## Abbreviations

ACJ: acromioclavicular joint
BMI: body mass index
DASH: Disabilities of the Arm Shoulder and Hand
n.c.: no change
n.s.: not significant
ROM: range of motion
RtA: return-to-activity
